# Post-disaster mental health and psychosocial support in the areas affected by the Great East Japan Earthquake: a qualitative study

**DOI:** 10.1186/s12888-019-2243-z

**Published:** 2019-08-27

**Authors:** Moe Seto, Harumi Nemoto, Natsuko Kobayashi, Saya Kikuchi, Nami Honda, Yoshiharu Kim, Ilan Kelman, Hiroaki Tomita

**Affiliations:** 1 Department of Disaster Psychiatry, Graduate School of Medicine, Tohoku University, Sendai, Japan; 2 Department of Disaster Psychiatry, International Research Institute of Disaster Science, Tohoku University, 2-1 Seiryo-Machi, Aoba-ku, Sendai, 980-8573 Japan; 3 Department of Psychiatry, Tohoku University Hospital, Sendai, Japan; 4 Department of Psychiatry, Graduate School of Medicine, Tohoku University, Sendai, Japan; 5 Department of Adult Mental Health, National Institute of Mental Health, National Center of Neurology and Psychiatry, Kodaira, Japan; 6 National Information Center of Disaster Mental Health, National Institute of Mental Health, National Center of Neurology and Psychiatry, Kodaira, Japan; 7 Institute for Risk and Disaster Reduction and Institute for Global Health, University College London, London, UK; 8 University of Agder, Kristiansand, Norway

**Keywords:** Disaster, Mental health, Psychosocial support, MHPSS, Long-term after a disaster

## Abstract

**Background:**

Few studies exploring the actual practices implemented for long-term mental health and psychosocial support after a natural disaster have been published. This study aimed to reveal (1) the types of activities that were actually provided as mental health and psychosocial support (MHPSS) in the long-term phase after the Great East Japan Earthquake (GEJE) and (2) the problems that must be addressed to provide post-disaster MHPSS activities.

**Methods:**

An open-ended questionnaire was sent to organizations in the Iwate, Miyagi and Fukushima prefectures that were potentially involved in providing MHPSS to communities affected by the GEJE. The organizations were asked to describe their activities and the problems that needed to be addressed to provide these support activities. The collected statements were analysed using content analysis with NVivo11.

**Results:**

The support activities conducted to provide MHPSS in the long-term phase after the catastrophe were diverse and classified into 7 major categories, namely, (1) one-on-one support for individuals in need of assistance, (2) support for collective activities, (3) support around living conditions and income, (4) increasing public awareness about mental health, (5) human resource development to improve response capabilities for MHPSS, (6) support for MHPSS providers, and (7) facilitating collaborations among the MHPSS activities provided to affected communities. Problems with human resources and funding were the most frequently mentioned concerns among the organizations participating in the survey.

**Conclusions:**

The establishment of systems to collect and share sufficient and relevant knowledge and to coordinate organizations for long-term post-disaster postventions would be desirable.

**Electronic supplementary material:**

The online version of this article (10.1186/s12888-019-2243-z) contains supplementary material, which is available to authorized users.

## Background

The Inter-Agency Standing Committee (IASC) guidelines on mental health and psychosocial support (MHPSS) in emergency settings describe the term ‘MHPSS’ as ‘any type of local or outside support that aims to protect or promote psychosocial well-being and/or prevent or treat mental disorder’. Mental health and psychosocial problems in disaster settings are interconnected, and the support for these problems are closely related and overlaping [1]. Examples of problems related to mental health include a pre-existing mental disorder and disaster-induced problems such as distress, grief, anxiety, depression, and post-traumatic stress disorder (PTSD). On the other hand, examples of problems related to psychosocial aspects include poverty that continues after the disaster, disaster-induced family separation, disruption of social networks, and destruction of community structures or traditional support mechanisms. Thus, MHPSS includes a wide range of activities with both biopsychiatric and non-medical approaches. Several other guidelines for MHPSS have been proposed, such as The European Network for Traumatic Stress (TENTS) Guideline for psychosocial care following disasters and major incidents published in 2008 [2], and the comprehensive guideline on mental health and psychosocial support in disaster settings published by the Operationalising Psychosocial Support in Crisis (OPSIC) Project in 2016 [3]. However, these guidelines have mainly focused on the acute phase after disasters; a consensus on the definition of the acute phase of a disaster is unavailable, but it is suggested to range from a week [4] to 3 months [5]. Only a few systematic studies of the activities that should comprise post-disaster MHPSS in the long-term phase, which lasts for many years after the acute phase, have been performed, although long-lasting MHPSS needs in post-disaster settings have been suggested based on the assessments of affected communities [6–13]. Some studies have investigated systematic support provided by mental health professionals (i.e., psychiatrists, psychiatric nurses, clinical psychologists, or psychosocial workers) for people suffering from mental health problems, and they discussed the contents of the support services (i.e., screening for common disorders after the disaster, assessment, and therapies such as cognitive behavioural therapy, medication management, and prescriptions), financial costs and human resources [14–16]. However, MHPSS can include a much wider range of activities provided by both mental health professionals and people who have not been trained as mental health professionals. Long-term MHPSS has rarely been systematically investigated in post-disaster settings.

The Great East Japan Earthquake (GEJE) occurred on 11 March 2011 underneath the ocean off the coast of Miyagi. The earthquake and subsequent tsunami killed over 15,000 people [17], and more than 300,000 people were evacuated to shelters, temporary housing, or the homes of relatives at the height of the crisis; some individuals were evacuated due to the nuclear power plant disaster caused by the earthquake [18]. Immediately after the disaster, a number of professional mental health care teams were organized all over Japan and travelled to the affected areas to continue to provide pre-disaster psychiatric services, treatments for acute stress reactions or psychoeducation at the shelters and temporary housing [19]. Based on the knowledge obtained after the Great Hanshin-Awaji Earthquake of 1995 and Niigataken Chuetsu-oki Earthquake in 2007, the long-term mental health and psychosocial needs were recognized [20–22]. As a result, a wide range of MHPSS activities was continued by governmental organizations, non-governmental organizations, universities, and other organizations after the acute phase.

The central government and the prefectural governments designed and built budgets for post-disaster MHPSS activities. The health sector of the municipal governmental organizations of the affected area were directly involved in providing MHPSS to the affected communities. Municipal governments also deployed support centres in most of the temporary housing complexes, which facilitate networking of the residents and support populations at risk of isolation. These efforts were based on the lessons learned from the post-disaster settings of the Great Hanshin-Awaji Earthquake in 1995, where many residents died alone [23].

As a type of permanent mental health care system, a mental health and welfare centre has been established in each prefecture since 1965, and public health centres are established in 8–10 districts of prefectures. These public organizations have played major roles in promoting the mental health of the community members, including patients suffering from psychiatric disorders, and coordination among the governmental and non-governmental organizations to improve the mental health of the communities. These organizations should have the primary responsibility of addressing the mental health of individuals residing in the communities affected by a disaster and have actually assumed major roles in coordinating related organizations in all previous disasters, particularly in the acute phase of the disasters. However, as an increase in MHPSS needs was recognized after the 1995 Great Hanshin-Awaji Earthquake, major non-governmental organizations, called “Kokoro-no Care Center” (referring to post-disaster MHPSS centres in Japan) were founded in Kobe 5 months after the disaster to provide long-term MHPSS. Thereafter, another “Kokoro-no Care Center” was founded in Niigata after the 2004 Niigataken Chuetsu Earthquake. Similarly, a year after the 2011 GEJE, three other “Kokoro-no Care Centers” were established in the Iwate, Miyagi and Fukushima prefectures and were modelled after the first two. The centres have been operated by a university or a non-profit organization funded by the reconstruction budgets from the Japanese government. Each “Kokoro-no Care Center” consists of approximately two dozen staff members, each of whom assumed major roles in providing MHPSS to the affected communities by coordinating with other organizations.

Other types of nonprofit organizations, such as “The Bridge of Mind of IWATE” in Iwate prefecture, “Kara Koro station” in Miyagi prefecture, “Mental Health Outreach Clinics Nagomi” in Fukushima prefecture, were founded as representative nonprofit organizations to provide MHPSS in the affected area. Unlike the “Kokoro-no Care Center”, these organizations are not funded by the government but are instead funded by financial resources such as donations.

In addition, many other non-governmental organizations were newly established or have become involved in MHPSS in the communities affected by the disaster. Post-disaster long-term MHPSS activities conducted after the GEJE have been too wide ranging to completely grasp and were not well connected or organized. This study aims to present an overview of these long-term MHPSS activities conducted in the communities affected by the GEJE and analyse problems that need to be improved to provide better long-term post-disaster MHPSS.

When initiating MHPSS activities, a useful tool would be to collect information about what types of MHPSS activities have been previously provided and the problems that must be addressed to provide post-disaster MHPSS activities. By sharing the knowledge of post-disaster MHPSS, providers can better understand the post-disaster MHPSS activities provided by other organizations, learn from them and facilitate collaboration with each other. Such information would help organizations improve post-disaster MHPSS. This study aimed to reveal (1) what types of activities were actually conducted with the intention of providing post-disaster MHPSS in the long-term phase after the GEJE and (2) what problems need to be addressed to continue and improve post-disaster MHPSS activities.

## Methods

### Data collection

Letters were sent to all of the non-governmental organizations in the Iwate, Miyagi, and Fukushima prefectures (*N* = 2108) registered at the Cabinet Office, Government of Japan [24] in December 2014, as well as the local government (*N* = 164) and educational facilities (*N* = 514) in the three prefectures to inquire whether they would participate in the survey by May 2015. Although a number of non-governmental organizations and educational facilities had been involved in MHPSS after the GEJE, a method to recognize which organizations had been involved in these activities was not available. Therefore, we sent letters to all of the organizations.

An open-ended questionnaire asked the organizations to describe (1) basic information about the organization, including the location, type of organization, the date of establishment, the time to initiate MHPSS activities after the GEJE, and if applicable, the time of termination of the activities; (2) the main activities of the organization; and (3) problems that needed to be addressed to improve their activities. Descriptions were returned via posted mail, fax, electronic mail, or a website formatted for answering the above questionnaire.

### Data analysis

The abovementioned information from the organizations was summarized to present an overview of the organizations involved in the study. The descriptions of the main activities of the organizations and problems that needed to be addressed to improve the activities were subjected to content analyses [25] using NVivo11 [26–28]. The definition of MHPSS or the range of activities covered by MHPSS is vague, and previous researchers have not investigated the problems among MHPSS activities that require improvement. The collection of information about the actual activities covered by MHPSS and extraction of the common problems and unique problems among the data would be beneficial. For data analysis, a content analysis was applied to the data to categorize the activities or problems and discuss the most commonly reported activities and problems. Content analysis is a systematic coding and categorizing approach that explores large amounts of existing textual data [29]. The key distinguishing feature of content analysis is that consistent sets of codes are used to designate data segments that contain similar material, and the frequencies of these codes are counted to determine the material contained in the data [30]. The descriptions were summarized into single sentences, and each sentence referred to a single post-disaster MHPSS activity or a single problem that needed to be addressed to improve the activity. Each sentence was defined as a single unit and subjected to coding. The reported problems regarding third parties were excluded, and only the problems regarding the respondents’ own organizations were subjected to the analyses. When the same activities were rephrased in multiple sentences, one of the sentences that most effectively described the essential factors of the activity was selected and the remaining sentences were discarded to avoid redundancies. When certain activities were depicted by multiple sequential sentences, these sentences were defined as a single unit. Units that shared similar content were classified into a single category, and the title of each category effectively representing the theme of the contents was defined. When applicable, some of the units that were classified into single categories and sharing closer content were classified into subcategories, and a title was also defined for each subcategory. The numbers of respondents were determined for each subcategory and category. This coding process was conducted by MS and verified by HT by reaching a consensus on the synthesized themes and subthemes. All data were collected and analysed in Japanese, and the results were translated into English for publication.

## Results

### Characteristics of organizations that provided MHPSS after the GEJE

Of the 2784 organizations to which we sent letters, 335 organizations returned the questionnaire. While 228 organizations were not involved in providing MHPSS after GEJE, 107 organizations provided descriptions of their activities related to MHPSS, as summarized in Table 1. Regarding the type of organization, 16 (15.0%) were public organizations funded by the local government, 17 (15.9%) were educational organizations, and 73 (69.2%) were nongovernmental organizations, including specified nonprofit corporations (nonprofit organizations that are authenticated) and general incorporated associations (a public interest organization that is registered). Thirty-seven organizations (34.6%) were newly established after the onset of the disaster. We sent letters to the representative contact and did not specify the role of the respondents. The respondents’ roles in their organizations were head of the organization = 38, administrative position = 30, head of the secretariat = 8, service provider = 8, and unknown = 23. There were no significant differences in the themes reported, regarding the activities conducted as MHPSS or the problems to overcome related to the provision of MHPSS, among the different types of organizations, probably because the majority of the responded organizations were nongovernmental organizations. Therefore, the types of organizations were not taken into account in the following analyses.

**Table 1:** Characteristics of organizations that provided MHPSS after the GEJE

	Number	Percent
Location of the organizations		
Iwate	23	21.5%
Miyagi	45	42.1%
Fukushima	39	36.4%
Type of organization		
Non-governmental organizations	74	69.2%
Governmental organizations	16	15.0%
Universities and other educational organizations	17	15.9%
The time of establishment		
Established before the GEJE	70	65.4%
Established after the GEJE	37	34.6%
The time to initiate MHPSS after GEJE		
Start		
Within 1 month after the GEJE	56	52.3%
Within 6 months after the GEJE	23	21.5%
Within 1 year after the GEJE	12	11.2%
Greater than 1 year after the GEJE	14	13.1%
Unknown	2	1.9%
End		
Within 1 year after the GEJE	16	15.0%
Within 2 years after the GEJE	3	2.8%
Within 3 years after the GEJE	1	0.9%
Greater than 3 years after the GEJE	1	0.9%
Ongoing (at the time of the survey conducted between December 2014 and May 2015)	86	80.4%

### Activities conducted as MHPSS for people affected by the GEJE

Two hundred forty sentences were analysed and classified into the following 7 categories using the content analysis [see the Additional file 1: Table S1].

#### (1) One-on-one support for individuals in need of assistance

Sixty-two units consisted of one-on-one-type support activities offered for individuals in need of assistance. The activities included a wide range of social work activities related to living conditions and employment, providing advice on mental health problems and physical health conditions (e.g., high blood pressure), providing advice on problems regarding maternal and child health and the development of children, and providing advice on fears regarding radioactive contamination due to the Fukushima nuclear power plant disaster.

- Outreach services

Thirty-seven support activities involved visiting the residences or activity sites of the affected people to “check up on the living and health conditions” of the residents and provide support to individuals in need. Residences included emergency shelter facilities, prefabricated temporary housing, private leases on housing with public financial support, and disaster public housing. Regarding the support for residences, the staff visited specific populations numerous times, including residents of prefabricated temporary housing, high-risk populations who were selected by a screening survey, people who were known to suffer from a mental disorder before the disaster, households with infants, and caregivers of orphans. Regarding the support provided in community spaces, free MHPSS, such as “monthly psychosocial case work” or “bimonthly maternal and child health consultations provided by clinical psychologists”, were offered.

One activity provided “free counselling services in public employment security offices or shopping malls”, to improve the accessibility to services for people in need. In addition, one activity offered counselling services that were “not restricted to the people severely affected by the disaster but also open to the people who themselves were not suffering but resided near the affected people, which resulted in the facilitation of interactions between these populations”. Regarding the one-on-one support for community activities, four support activities provided counselling during the regular activities of the organizations, as represented in the statements: “We offered hand massage or thermal massage in community spaces of prefabricated temporary housing. During the massages, we attentively listened to the residents. They seemed to feel that they could easily speak out in these somewhat intimate situations”, and “We intended to listen attentively to the affected people during programmes for exercises, a chat over tea, or nail polishing.”

b. Provision of stable spaces or pipelines for counselling

Eighteen support activities were designed to establish stable spaces or pipelines for counselling regarding mental health. One group reported “we established a pipeline specific for counselling on problems related to domestic violence or sexual violence, based on the lessons from previous disasters, in which women agonized with these problems.” One activity was designed to provide counselling services after regular service hours, during evenings and weekends.

c. Telephone-based counselling

Seven support activities offered “telephone-based counselling” for people suffering from mental health problems or fear of radioactive contamination due to the Fukushima nuclear plant accident. One activity established “a telephone number specific to the people suffering from the disaster”. One activity provided “telephone counselling services during nights and weekends”.

#### (2) Support for collective activities

The category ‘Support for collective activities’ consisted of 97 support activities, which was the largest number of units among the 7 categories.

- Organization of events to facilitate social interaction and prevent isolation

Of the 97 support activities, 45 support activities were selected as directly organizing events offered for affected residents. These events provided residents an opportunity to gather with surrounding people and enjoy some activities represented in descriptions such as “We held various kinds of circle activities for exercise, music, or handicrafts” or “regular casual meetings”, which aimed to facilitate social interaction among residents and prevent isolation in the situation of evacuation or temporary housing. In some activities, “dialect or folk songs were featured in the events” in an effort to facilitate the acceptability of the event by the communities.

b. Assistance for resident-initiated activities

Twenty-one support activities were described as assistance for the activities that were mainly provided by the affected residents themselves. Support for developing a self-government association for the newly reconstructed community or preserving the memory of the disaster in affected communities was provided in an effort to reinforce local activities. These activities are described by the statements listed below. “A memorial ceremony was held as a collaborative effort of local community members and members of an academic institute.” “We supported the management of social meetings among affected community members, otherwise community members were unable to easily manage the meetings by themselves.” “We supported a local community aiming to locate single elderly individuals in the community.” “We supported editing a local history of the affected area, where the townscape was drastically changed by the tsunami disaster. The recording of the history and the culture of the community was successful and helpful in that it served as a reminder of what members of the community had known and experienced prior to the tsunami.” “We assisted a local government in editing a town journal by interviewing community members and focusing on the landscaping of their homeland before the disaster. The journal has been used in elementary schools in the area to transmit the memory to the next generation.”

c. Provision of places and opportunities for play, learning or rest

Nineteen groups provided playgrounds for children or opportunities to exercise in the community spaces of prefabricated temporary housing facilities. These activities included “yoga”, “weight-lifting exercises”, “radio exercise” (rhythmic exercise routines set to music aired by the public broadcaster NHK, which is popular among Japanese communities), and “hiking”. In areas where children were unable to play outside due to the influence of the nuclear plant accident, 6 groups provided “places where children can play without worrying about anything” at indoor locations or areas away from the epicentre. Seven groups provided evacuated students “support for learning school subjects” after school or during a long vacation. Two groups provided “places where people with disabilities and their family members could stay for a while to take a break from away their residences after evacuation, where they might feel free about surrounding people to some extent”. Three other groups provided “places and opportunities to stay for a while in places away from the epicentre of the nuclear plant accident, where people felt free from anxiety about radiation”. One of the latter groups provided “a free shuttle service” for transportation in addition to the lodging service.

#### (3) Support around living conditions and income

Ten activities provided support around living conditions and income. For example, three groups supported “creating employment for people with disabilities and facilitated the sale of relevant merchandise”. Another group supported “facilitating the sale of handicrafts” made by elderly residents in prefabricated temporary housing. While performing these activities, the residents often gathered in community spaces and spent time communicating with each other. Additionally, seven groups listed “the provision of meals” and “distribution of supporting supplies”.

#### (4) Increasing public awareness about mental health

Twenty-three support activities were conducted with the intention of increasing public awareness about mental health.

- Talks and seminars for the general population of the affected communities

Sixteen groups reported that they had provided “talks” and “seminars” for the general population of the affected communities. The topics covered “alcohol-related problems”, “mental health of the elderly or children”, and “fear of radioactive contamination” due to the Fukushima nuclear plant accident. One group said “we planned events based on the outcomes of questionnaire surveys to identify the needs of the affected residents”.

b. Publication and distribution of brochures covering topics related to mental health

Seven groups published and distributed brochures covering topics related to mental health, such as “techniques for relaxation”. One group published brochures covering resources and contact information for counselling on mental health and psychosocial problems. One group published “a column in existing public information papers and on homepages to provide information related to mental health”. One of the groups issued a file of record sheets entitled “A file for maintaining mental and physical health” to record the health conditions and development of children, in addition to the contact information for counselling about mental health problems or fear of radioactive contamination, thus ensuring that the affected individuals could maintain access to the information. One of the groups reported, “We set up a booth providing information to improve the awareness of mental health and held public health check-ups to attract the attention of people who cared about their physical health.”

#### (5) Human resource development to improve response capabilities for MHPSS

This category consisted of 23 units, which were the activities described by a wide range of MHPSS providers to improve the MHPSS skills of affected residents. The MHPSS providers included mental health professionals or other interpersonal service professionals. The activities also included non-professional personnel, such as the staff of a temporary housing support centre (who were typically hired from among affected community members to support other affected members), or local welfare commissioners (who volunteered to support community members and were assigned by a local government under the Consumer Committee Act), or even lay people who intended to become new MHPSS providers.

- Developing the skills of the MHPSS providers

Eighteen groups provided training for MHPSS providers who support disaster victims to improve support skills, such as “communication skills”, or “response skills for alcohol-related problems”. One group dispatched advisors and another group held “case study meetings to increase MHPSS providers’ skills”.

b. The development of local human resources

Four groups provided workshops entitled “a seminar to train gatekeepers for suicide prevention” or “training courses for mental health supporters” for the general population to foster mental health support in the community. One group offered practical training for the graduate students of a clinical psychology course to learn support skills for disaster victims in the affected areas.

#### (6) Support for MHPSS providers

The category ‘Support for MHPSS providers’ consisted of 15 units.

- Mental health care for MHPSS providers

Nine groups provided mental health support for MHPSS providers in the affected communities by “implementing mental health surveys”, “individual counselling” or “workshops”. Through these activities, opportunities for physical and mental care and information and skills for self-care were provided for MHPSS providers. One group said that “We have realized the importance of supporting staff who support people in the communities, and based on our experience, we have actually supported the staff.”

b. Dispatching human resources to organizations with limited personnel or expertise in mental health or psychosocial problems

Six support activities empowered the organizations to provide mental health support to affected people. Two groups reported that they “dispatched human resources” to the organizations in which the work volume was increased after the disaster, and 4 groups reported that they “dispatched mental health specialists, such as clinical psychologists or psychiatric social workers, to provide expertise to address mental health problems that developed after the disaster”.

#### (7) Facilitating collaborations among the MHPSS providers in affected communities

The category ‘Facilitating collaborations among the MHPSS providers in affected communities’ consisted of 10 units.

- Coordination of human resources and activities

Six groups reported that they provided “coordination of volunteers and MHPSS providers” who visited the affected area. These groups linked the personnel to organizations who needed additional staff or coordinated activities conducted by multiple organizations.

b. Providing opportunities to share information among related organizations

Four groups established places to share information and facilitate connections among MHPSS providers or among organizations that provided MHPSS to communities affected by the disaster. One of the groups reported that “At the meeting, they shared their experiences with sympathy, discussed their current situations and problems with their activities, and shared ideas to address the problems.”

### Problems to overcome related to the provision of MHPSS to people affected by the GEJE

One hundred one sentences were analysed and classified into the following 7 categories using the content analysis.

#### (1) Human resources

Twenty-nine units consisted of problems with human resources. This category contained the largest number of units among the 7 categories

- Shortage of personnel in general

Twenty-four groups reported the shortage of personnel, as represented in the following statements: “We have a shortage of human resources to conduct activities in response to the demands from the communities” and “Because of difficulties in hiring enough staff, we were unable to provide as many support activities as we expected.”

b. Shortage of people with specialized support skills

Four groups reported the shortage of people with specialized support skills, as represented in statements: “Because we do not have any staff with specialized support skills, we cannot provide long-term support for people who need specific types of assistance” and “Staffs potentially lack the opportunities to be trained to become more skilful and to assume responsibility for and improve the activities.”

c. Instability of employment

One group reported that they had “difficulty securing human resources” because they operated with a single-year budget, and therefore, they were able to hire staff with only a single year contract. The unavailability of long-term employment contracts hindered the organization’s ability to attract new staff and people who could develop skills related to supporting the affected populations.

#### (2) Funding for MHPSS

The category ‘Funding for MHPSS’ consisted of 28 units, which described problems regarding securing financial resources for operating activities and labour costs.

- Limited financial resources

Twenty-seven groups claimed that they were not able to obtain sufficient funding to conduct their support activities. Four of the 27 groups described that they had difficulty in maintaining the continuity of support activities due to limited funds, as represented in the statements listed below. “Our activities are unstable because there is no guarantee for continuity in economic support in subsequent years” and “We have provided support activities in the affected areas based on budgets provided by numerous funding agencies, but many of the agencies will likely terminate the economic support soon. A serious problem is to secure stable financial resources.” Another 4 groups described that their activities were limited due to a lack of funds, which is represented as follows: “We would like to provide many services to the affected people, but we had to abandon many potential effective activities due to the budget shortfall.” Along with the shortage of human resources, the shortage of financial resources also limited their support activities.

b. Mismatch between subsidies and needs

The nonprofit organizations had problems in using subsidies as the main financial resources for their activities, as represented by the following statement: “Gaps exist between what the funding agencies intended to support and what is really needed in the affected communities.” The target area of subsidies must be expanded.

#### (3) Strategies and skills for MHPSS

Twenty-six groups reported problems regarding strategies and skills for MHPSS for residents in the affected area.

- Limited number of staff with specialized support skills

Regarding overall support skills, five groups reported that the limited number of staff with specialized support skills caused confusion and a lack of confidence in their support activities, as represented by the following statements: “We are not certain whether we can provide sufficient care” and “We have no staff who are specialized in counselling, and thus we are worried about how to provide support in those situations in which residents’ problems were widely varied.” One of the groups reported limited opportunities for training to improve support skills, as represented by the statements provided below. “We have learned the attentive listening skill to some extent through relevant workshops. However, we have not been able to easily manage time and provide trainers to train ourselves sufficiently.” Regarding the setting of goals for MHPSS, three groups said that they experienced difficulty in setting goals for MHPSS, as described below. “We were not sure what types of situations we should aim to address using MHPSS activities” and “We could not easily estimate how long we would succeed in providing our support activities.” In addition, regarding the assessment of needs, three groups reported that MHPSS was difficult to provide to the people who needed the support. One of the groups claimed that they were not allowed to access information about the affected residents with potential needs for MHPSS. This information should have been accumulated by the local government, and only a limited number of organizations with a contract were able to share information between local governments and the organizations for post-disaster MHPSS that was outsourced from the local government to other organizations. Furthermore, regarding the difficulty in approaching people who are likely to need but do not seek support, two groups said that they had problems in determining the best method to approach these individuals. The majority of the organizations provided support activities in response to requests, with several notable exceptions, such as providing outreach to residences. These concerns are represented in the following statements: “We were concerned about how to provide support and information to people who were in a situation of social withdrawal” and “Our group’s problem was how to support people who did not participate in events held by support groups.”

b. Difficulty in locating sites for MHPSS activities

Four groups said that they “struggled to secure sites” for their activities. One of the groups reported that they “located and negotiated to secure places” to provide support activities by themselves. The group proposed that “an ideal situation would be a central organization that matched support organizations that intended to provide certain types of support activities and sites or people with certain types of needs to be supported by comprehending the entire situation”. Two groups stated problems with accessibility to support due to physical or mental distances, as represented by the statements listed below. “The affected area was too extensive. Therefore, even if a counselling facility was available in a certain place, the people who needed support might not be able to reach the facility” “A friendly atmosphere where residents feel free to consult or ask for support would better be facilitated.”

c. Difficulty in continuing support activities

Three groups mentioned difficulties in continuing to provide support to affected residents, as represented by the following comments: “Support activities are difficult to continue, and we need to develop our support activities as a permanent regional support program.”

d. Difficulty in communicating risk associated with radioactive contamination

Two groups reported that they managed to provide support related to the fear of radioactive contamination, and experienced difficulty in collecting information about how to support the people, as represented in the statements listed below. “We did not know which information regarding radioactive contamination was accurate among the various forms of available information. We listened to residents’ anxieties and opinions without denying them, and shared new information with everyone as much as possible. We also attempted to obtain expertise in providing the best response at the moment. However, it took quite a lot of time and labour to provide those activities, and we do not know whether our support strategies were effective.”

e. Limited numbers of comprehensive supports

Two groups identified the necessity of linkages between supports for general living conditions and mental health, as represented by the statements listed below. “We believe that the effectiveness of MHPSS is limited unless it is linked to support for reconstruction of the residence and community” and “A life without a clear future is likely to result in great anxiety, which also increases the risks of mental health problems.”

#### (4) Difficulty in presenting and documenting their achievements and the cost-effectiveness of MHPSS activities to funders

Five groups reported difficulty in presenting their achievements and the cost-effectiveness of their MHPSS activities. Three groups stated that they experienced difficulty in presenting their achievements, as represented in the following statement: “Because we do not have a person who is good at analyses, such as a researcher, we are unable to easily show our achievements in an objective manner, for example with figures.” Two groups noted difficulties in showing the objective cost effectiveness of their support activities that were related to obtaining funds for their activities, as represented by the statements listed below. “In most cases, we are expected to show cost effectiveness before obtaining financial support, but it is difficult to prove with evidence.” “Organizations providing MHPSS activities note that it is quite difficult to prove the effectiveness of their activities with figures, making it harder to obtain a budget.”

#### (5) Insufficient care for the MHPSS providers themselves

Four groups noted a need for care for their staff, as described in the statements listed below. “We need mental health care for our staff who have been involved in providing MHPSS activities. Because some of our supporting members have not been trained as specialists, they themselves are sometimes affected by the situation.” “Our staff suffer unless they receive proper cared; however, we experience difficulties in providing mental health support for our staff.” “Long-lasting care for staff who provide MHPSS for affected residents is necessary; otherwise, the staff will eventually be worn out or burned out, and it will damage the body of organizations providing MHPSS.”

#### (6) Difficulty in balancing between the MHPSS activities and normal activities

Two organizations that existed before the disaster reported conflicts in balancing their regular activities and post disaster interventios for the affected area, as described in the statements listed below. “Our organization had a full schedule of regular work, and were not able to provide post-disaster support activities for the outside area.” “Because the timing of regular activities overlapped among the members, we were unable to provide support during the summer holidays when support was needed.”

#### (7) Cooperation with other organizations

Seven groups identified problems in cooperation with other organizations. The seven organizations reported that they felt they felt insufficient information was shared with other relevant organizations.

- Insufficient cooperation and coordination with other organizations

Five groups noted insufficient cooperation as follows: “Sufficient mutual communication was not available between support organizations. More sharing of information would have enabled us to provide more support to the people needing it” and “Cooperation and coordination among related organizations would be desirable to maximize the benefits from very limited resources.”

b. Lack of systems to cooperate and coordinate MHPSS activities provided by various organizations

Two groups reported that although cooperation was necessary, a system designed to arrange cooperation in meeting the affected individuals’ needs was not available. A long time was needed to merely build the foundation of each activity, and may impose a burden on each organization to build cooperation in addition to their own activities for the affected residents and communities. This problem is represented in the following statement: “We went to the afflicted area and spent a year communicating with the community. In the community, we expended a great deal of effort to locate people at high risk from the mental health perspective, and we were not able to manage the time and effort to build a bridge with related organizations. No system was available to facilitate cooperation.”

## Discussion

### Activities conducted as MHPSS for people affected by the GEJE

This study profiled the wide range of MHPSS activities conducted by various organizations after the GEJE. While 27 organizations (20%) terminated their activities within 2 years after the GEJE, eighty-six (80%) reported ongoing activities at the time of the survey, which was conducted between December 2014 and May 2015. The data indicated that the majority of organizations maintained long-term MHPSS activities 3–4 years after the disaster, although the data might be affected by a sampling bias that organization which provided support activities only in the acute phase may have had less motivation to respond to the inquiry.

Following the IASC guidelines describing the MHPSS as all activities from inside and outside of the affected community designed to protect and promote psychosocial wellbeing, or prevent and treat a person with mental illness [1], previous studies have considered that post-disaster MHPSS should not exclusively focus on support to improve mental health conditions of people with mental disorders [31]. On the other hand, very few papers have discussed the actual MHPSS activities provided to communities affected by a disaster. Only a few articles have reported the provision of mental health support by professionals to high-risk populations [14, 15], probably because the organizations involved in providing post-disaster MHPSS are so varied that a grasp of the whole picture is difficult.

Thus, the content analysis performed in the current study first characterized activities conducted as MHPSS in the long-term phase after the disaster. The analysis was based on a comprehensive survey of activities provided in the three major prefectures affected by the GEJE, and classified into 7 major categories. The data endorsed the theoretical knowledge presented in the previous literature and indicated the wide range of support that is needed to improve the conditions of people who are at risk of suffering from mental health and psychosocial problems due to their exposure to difficulties related to the disaster. Although categories more frequently reported by the organizations were emphasized by being listed at first, discussions were made for all categories because the remaining categories also seem to include meaningful information.

One-on-one support for the individual needs of the affected people provided by the majority of organizations that participated in the study as MHPSS activities was mainly based on outreach. The result was consistent with the basic principles of mental health in emergencies proposed by van Ommeren in 2005 that outreach and awareness programs are important to ensure the treatment of vulnerable groups [31]. On the other hand, none of the organizations investigated in the current study reported that they were directly involved in providing medical treatment to patients with mental health disorders, such as depression or PTSD, after a disaster, probably because medical facilities, such as hospitals or clinics, were not included as subjects of this study. In Japan, at least in the long-term phase after a disaster, medical treatment is provided by medical facilities and is not tightly incorporated into a system for MHPSS, although some of the subjects who received MHPSS were referred to medical facilities. This condition contrasts with cases in the US, in which medical treatment seems to be included in a system for postventions of affected communities [15].

Support for collective activities also was a major type of MHPSS, and activities included in this type of support varied widely. Different organisations may have had different mandates, and thus diverse MHPSS activities were available that potentially suited different needs. Many of these organizations aiming to provide MHPSS to communities affected by the disaster conducted activities to improve the living conditions by obtaining income or supplies, or to secure opportunities to participate collective activities or daily activities (e.g., play, exercise, and learning after school) designed to facilitate adaptation and independence in reconstructed daily life as MHPSS activities. The importance of providing these supports was previously suggested by the TENTS Guideline for psychosocial care following disasters and major incidents [2], which denotes that “work/rehabilitation opportunities should be provided to enable those affected to re-adapt to daily life routines and be independent” as an problem to be considered in periods exceeding 3 months after the disaster. In addition, the problem is related to the concept of ‘Child Friendly Spaces’ in the Guidelines For Child Friendly Spaces in Emergencies, which emphasized children should be provided opportunities and spaces for play or learning in emergency settings [32]. Notably, many of the organizations that participated in this survey considered these supports for living conditions as MHPSS activities. The provision of meals and distribution of supplies as support for living conditions were included in acute phase MHPSS [33]. Based on the findings from the current study, needs for these activities may persist for years after a disaster occurs. In the long-term phase after the disaster, these activities may be emphasized not only for securing better living conditions but also as opportunities for enhancing the mental health conditions of the affected populations.

In the current study, we systematically investigated the long-term MHPSS activities that were actually conducted in the communities affected by the GEJE. Most of the MHPSS activities that were conducted several years after the GEJE have been suggested as MHPSS in previous guidelines and publications. For example, the importance of increasing public awareness about mental health was highlighted by Juen, B et al. [3], the importance of providing training and supervision for MHPSS providers in emergency settings was described by van Ommeren et al. [31] and the IASC guidelines [1]. Because these previous guidelines and publications were mainly based on the acute phase after a disaster, many of these activities that were conducted immediately after the disaster were also beneficial in the long-term phase after a disaster while affected individuals were adjusting to the culture and reconstruction of the area. In addition, the study indicates a need for increasing awareness and training that draws on past experiences and enables the application of existing knowledge and expertise.

### Problems to overcome in the MHPSS activities designed for people affected by the GEJE

The content analysis identified 7 categories regarding problems in post-disaster MHPSS activities that must be addressed: human resources, funding for MHPSS, strategies and skills for MHPSS, difficulty in presenting and documenting their achievements and the cost-effectiveness of MHPSS activities, insufficient care for the MHPSS providers themselves, difficulty in balancing between the MHPSS activities and normal activities, and cooperation with other organizations.

Problems with human resources and funding were the most commonly shared concerns among the organizations that participated in this survey. Human resources and stable funding have been identified as important problems in MHPSS in several previous studies [3, 15, 34]. These two problems are theoretically related to each other and may consist of the major factors underlying the difficulty in managing and continuing the activities. Shoenbaum et al. calculated that the costs of mental health care providing screening and treatment for the residents affected by hurricanes Katrina and Rita were $1133 per capita or a total of $12.5 billion [15]. Subsequently, this estimate was proposed indicate a scope that is far larger than most post-disaster psychiatry responses intend to assume, but it does provide a perspective of the magnitude of the resources that are potentially needed to “do things right” [35]. In addition to continuing the top-down support for people who are at high-risk, as suggested in the proposal by Schoenbaum, the provision of financial support for organizations that promote bottom-up MHPSS might be beneficial, depending on potential social resources available for MHPSS in the local communities affected by a disaster.

Difficulty in setting goals and MHPSS skills training was also a concern noted by many organizations. Strategies designed to address the difficulty in setting goals and providing MHPSS skills training may be a feasible solution. An important task is to accumulate knowledge and intellectual resources, and then establish a system to share them efficiently among the relevant organizations and societies, which will not only solve the third problem but also conserve the necessary human resources and funding. The lack of goals and training in MHPSS skills may result in insufficient disaster preparedness in terms of disaster-related mental health. A plan for long-term post-disaster MHPSS and the ability to learn basic skills for providing support are necessary before a disaster occurs, i.e., in ordinary settings. Advance planning for the management of the organization conducting MHPSS should include the plans for financial and human resources. TENTS [2] and OPSIC [3] guidelines also recommend that governments/authorities should provide adequate funding to maintain an appropriate psychosocial care plan that is able to be effectively delivered should a disaster occur, and MHPSS providers (both professionals and lay volunteers) should be recruited in advance.

Strategies designed to resolve the difficulty associated with the fourth concern, difficulty in presenting their achievements and the cost-effectiveness of MHPSS activities, also represent a feasible solution for the problems associated with funding and human resources. According to Dückers et al., occasional evaluations of a support activity are helpful to address the local needs and to promote the implementation of MHPSS [36]. The monitoring and occasional evaluations of MHPSS activities would be beneficial for providing feed back to the publishers of guidelines for updates. These evaluations would also be helpful for funding agencies and organization themselves, if the organizations were able to objectively verify the effectiveness of their activities and plan their subsequent activities based on the evidence. Moreover, this evaluation would be advantageous for obtaining and maintaining funding. The promotion of cooperation between academic research institutes and supporting organizations represents a useful solution to solve the fourth problem, and potentially the first two problems. Additionally, each group must be aware of the need to create an activity plan and activity report and to generate records of their activities, which would facilitate the accumulation of knowledge. Furthermore, a common format that integrates key items for planning and reporting of the support activities should be generated and shared among the communities, and common. Systems to archive those plans and report on supporting activities could be developed for sharing information among societies over time.

Insufficient care for the MHPSS providers themselves and difficulty in balancing between the MHPSS activities and normal activities were also listed by many organizations. The importance of self-care for MHPSS providers is emphasized in the guidelines focused on the acute phase of post-disaster MHHSS, while it seems to be less prioritized in the long-term MHPSS activities. In the acute phase, MHPSS providers tend to reach out to the communities affected by the disaster based on the prepared systems for disaster response and work during a restricted period, focusing on the MHPSS. On the other hand, in the long-term phase, a wide range of local populations participate in ongoing MHPSS activities. Many of these organizations must balance the MHPSS activities and normal activities, and the boundaries between MHPSS and ordinary activities tend to be obscure.

Strategies designed to address the seventh concern, insufficient cooperation with other organizations and the facilitation of cooperation, also represent feasible solutions for the first two problems. While human resources and sufficient funding to maintain each organization and its activities might be difficult to obtain, sharing information and resources, such as training or specific support activities, could improve this situation. The lack of systems to facilitate cooperation among the relevant organizations for long-term post-disaster support was noted. A worthwhile solution is the establishment of a system with a hub responsible for sharing information and coordinating activities among relevant organizations. The unification of formats of reports with other organizations would facilitate the aggregation of information, including present situations and problems remaining to be addressed, which would enhance the collaboration and sharing of resources.

If systems were designed to accumulate and share sufficient knowledge and to coordinate organizations providing long-term post-disaster MHPSS, they would reduce the burden for establishing activity bases by facilitating cooperation, instead of merely depending on the effort and knowledge of each organization. These problems were identified in a previous report discussing methods to address mental and social health during and after acute emergencies, e.g., “Strong collaboration with other support agencies will avoid wastage of resources” or “Continuous involvement of the government, local university or established local organizations is essential for sustainability” [31]. The current study endorsed these insights with actual statements from support organizations who claimed that these collaborative relationships are needed but have not yet been established.

### Limitations

This study had several limitations. First, the sample of 107 organizations providing MHPSS might not represent the total activities, because we were unable to easily identify the total number of organizations that provided post-disaster MHPSS after the GEJE. Second, we were not able to clarify the detailed contents of one-on-one support. Furthermore, since the survey of medical institutions was conducted in another study, we were unable to determine the supports available to connect people in need of treatment for conditions such as PTSD from outreach services.

## Conclusions

In conclusion, many organizations conducted a wide range of post-disaster MHPSS activities in the long-term phase after the GEJE, suggesting that different organizations have different mandates and diverse MHPSS activities were available, potentially suiting different needs. At the same time, various problems were addressed in the management of MHPSS activities. Most of the activities and problems associated with providing MHPSS had been suggested in existing guidelines and publications, indicating a need for increasing awareness and training about available materials to draw on past experiences and to apply existing expertise. Advance planning by organizations to conduct long-term MHPSS activities and considering financial and human resources would assist in resolving this problem. Strategies designed to monitor and evaluate the effectiveness of MHPSS activities should be established, particularly for providing feed back to the publishers of guidelines for updates. The establishment of systems to coordinate multiple relevant organizations and to accumulate and share experience and knowledge of MHPSS activities may be desirable, with a particular focus on obtaining a better understanding of the implications of incorporating a wide range of organizations and activities.

## Additional file

Additional file 1: **Table S1.** Activities conducted as mental health and psychosocial support after the Great East Japan Earthquake. The table summarizes 240 sentences regarding mental health and psychosocial support activities reported by the 107 organizations, which were classified into seven main categories using the content analysis. (XLSX 1209 kb)

## Data Availability

Detailed data are available for access upon request. In those cases, some of the information will be excluded from the raw data to protect personal information.

## Abbreviations

GEJE: Great East Japan Earthquake
IASC: Inter-Agency Standing Committee
MHPSS: Mental Health and Psychosocial Support
OPSIC: Operationalising Psychosocial Support in Crisis
PTSD: Post-traumatic stress disorder
TENTS: The European Network for Traumatic Stress
